# Quantitative regulation of Waxy expression by CRISPR/Cas9‐based promoter and 5'UTR‐intron editing improves grain quality in rice

**DOI:** 10.1111/pbi.13427

**Published:** 2020-06-18

**Authors:** Dongchang Zeng, Taoli Liu, Xingliang Ma, Bin Wang, Zhiye Zheng, Yaling Zhang, Xianrong Xie, Bowen Yang, Zhe Zhao, Qinlong Zhu, Yao‐Guang Liu

**Affiliations:** ^1^ State Key Laboratory for Conservation and Utilization of Subtropical Agro‐Bioresources Guangzhou China; ^2^ Guangdong Laboratory for Lingnan Modern Agriculture Guangzhou China; ^3^ College of Life Sciences South China Agricultural University Guangzhou China

**Keywords:** genome editing, CRISPR/Cas, rice, amylose content, *Waxy*, quantitative trait

In cereal crops, grain starches are composed of different proportions of amylose and amylopectin, which determine the cooking and eating qualities. The amylose synthesis is controlled by the *Waxy* (*Wx*) gene encoding a granule bound NDP‐glucose‐starch glucosyltransferase (Shure *et al*., 1983). In rice (*Oryza sativa* L.), the varied activities of natural *Wx* alleles regulate different amylose contents (AC), gel consistency (GC) and pasting viscosity of grain starches; these factors together influence the grain appearance, cooking/eating quality and starch physical characters (Zhang *et al*., 2019). *Wx^a^* is a strong allele mainly distributing in *indica* (an *O. sativa* subspecies) cultivars producing high ACs (25%–30%) (Wang *et al*., 1995). While *Wx^b^*, presenting mainly in *japonica* (another subspecies) cultivars, is a weak allele producing moderate ACs (15–18%) (Isshiki *et al*., 1998). Generally, rice grains with higher ACs and lower GC values have poor eating quality, while those with moderate ACs (15–20%) and higher GC values (60–80 mm) give better taste for most consumers. Using the successive backcrossing methods, *Wx^b^* can be introgressed into *indica* varieties to improve the grain quality. However, the traditional breeding methods are time consuming and difficult to break close linkage drags with undesirable traits.

We previously employed CRISPR/Cas9 to target the *Wx* coding region to generate glutinous rice (Ma *et al*., 2015). However, this kind of function‐knockout strategy produces only null gene alleles, and when *Wx* is targeted generally glutinous lines are generated. Studies on generating various quantitative variations of traits by genome editing are rare. To rapidly improve rice grain quality, here, we developed CRISPR/Cas9 editing strategies to generate new *Wx* alleles producing various ACs by quantitative regulation of its expression, using an elite *indica* variety TianFengB (TFB) as a test. TFB is a widely used parent in hybrid rice breeding for its high‐yield performance, but its grain quality (and of the resultant hybrids) is poor due to higher AC (ca. 25%) and lower GC (56 mm; see below).

Disruption of promoter sequences by genome editing may change agronomic traits (Li *et al*., 2017; Rodríguez‐Leal *et al*., 2017). Therefore, we selected a ca. 2.0‐kb upstream sequence of *Wx^a^* in TFB for targeting, which contains a 0.9‐kb promoter regulatory region and a 1.1‐kb intron‐containing 5’untranslation region (UTR) (Figure 1a). The first strategy is based on transcriptional regulation, thus we analysed the promoter sequence using Plant‐CARE (http://bioinformatics.psb.ugent.be/webtools/plantcare/html/) and identified three putative *cis*‐regulatory elements (CREs), Endosperm‐box, A‐box and CAAT‐box. We designed four pairs of targets (T1–T8) in this region (Figure 1a) using CRISPR‐GE (Xie *et al*., 2017) for multiplex editing. The second strategy we explored is for post‐transcriptional regulation by targeting the 5’UTR intronic splicing site (5’UISS) of *Wx^a^* with a target T9 to alter the intron‐splicing pattern and efficiency. In addition, a coding‐exon editing (with a target T10) was done to produce glutinous rice. Using our CRISPR/Cas9 system (Ma *et al*., 2015), we prepared six constructs for the double‐target or single‐target editing (Figure 1a, b), and used them for *Agrobacterium*‐mediated transformation of TFB.

**Figure 1:**
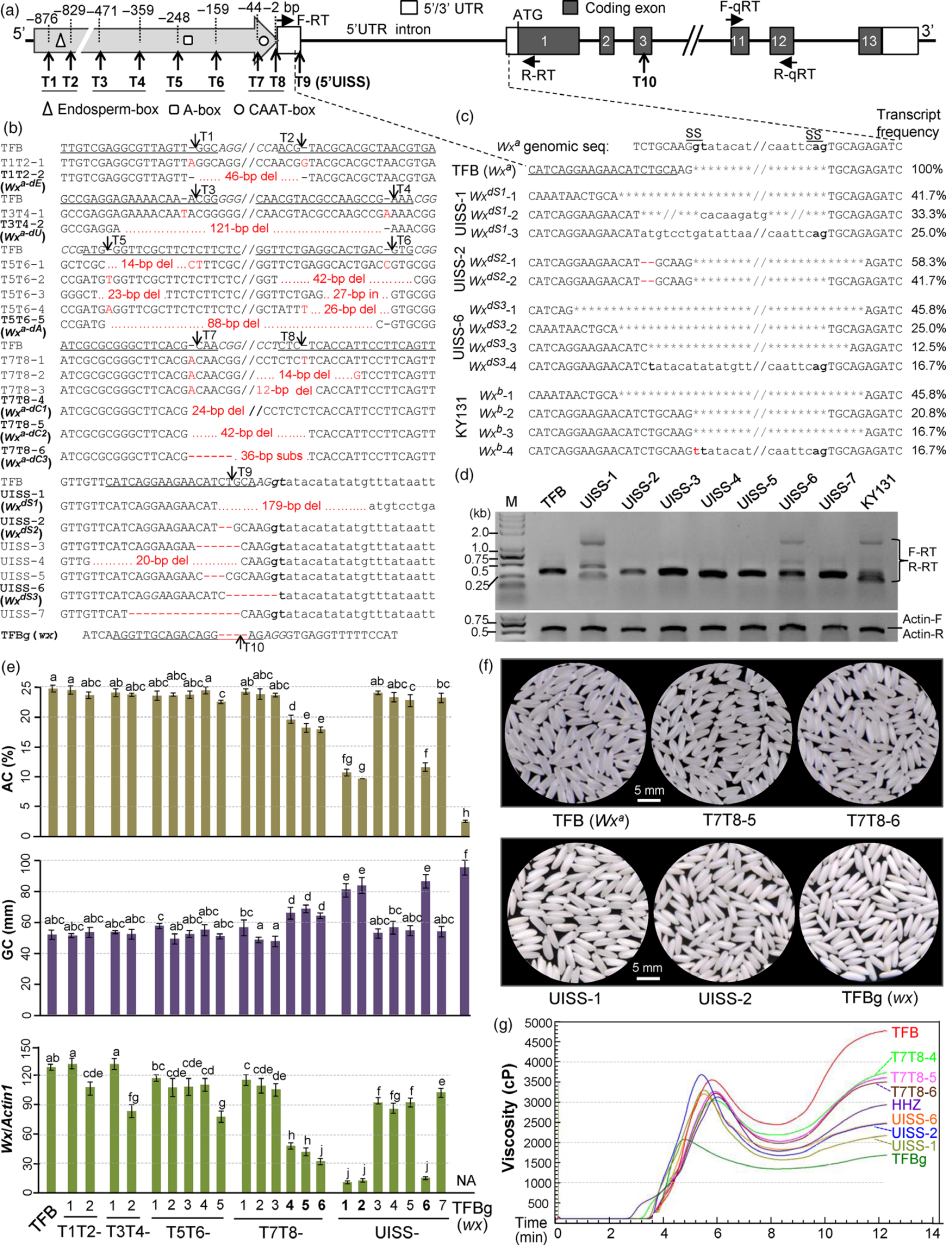
Improvement of rice grain quality by quantitative regulation of *Wx* expression via promoter and 5’UISS editing using CRISPR/Cas9. (a) Structure of *Wx^a^* and target sites at the promoter region (T1–T8 in four pairs), the intronic splicing site within the 5’untranslation region (5’UISS; T9) or a coding exon (T10). F‐RT/R‐RT and F‐qRT/R‐qRT, primers for RT‐PCR and qRT‐PCR, respectively. (b) Nucleotide variations (in red) at the targets (protospacer adjacent motif in italic) of homozygous mutant lines (T_2_) from an *indica* variety TFB carrying *Wx^a^*. ‘‐’ and ‘del’, base deletion; ‘in’, base insertion; ‘subs’, base substitution (AGACACAAATTCCTTCAGTTCTTTGTCTATCGGGCT). Sequences between the targets are omitted. Lower‐case letters, the intron. TFBg, a glutinous line. (c, d) cDNAs (RNAs from 15‐day‐old seeds) showing mRNA splicing by sequencing 24 clones each line (c) and agarose‐gel analysis (d). Asterisks indicate spliced‐out nucleotides. KY131, a *japonica* variety with *Wx^b^* having a G‐to‐T mutation at the splicing site (SS). *Actin 1*, a control. (e) Measurements of *Wx* expression, amylose content (AC) and gel consistency (GC). Bars, SD (*n* = 3). Samples without a same letter show significant difference by Duncan's test (*P* < 0.05). (f) Polished grains of TFB and representative edited lines. (g) Rapid visco analysis profiles of grain starches of the lines. HHZ, an *indica* variety (with *Wx^b^* and 17.1% AC) as a comparison. cP (centi Poise), viscosity unit.

From transgenic (T_1_) segregating families, we PCR‐selected transgene‐free plants and further identified 23 homozygous mutant T_2_‐lines (Figure 1b). These lines had base insertions or deletions at the targets, or fragment deletions (or a 36‐bp substitution) between the paired targets, which removed the putative CREs, respectively (Figure 1b). In two lines (UISS‐1, UISS‐6) by the 5’UISS‐editing, the intronic splicing site (GT) was deleted (Figure 1c). We named some of these *Wx* mutant alleles that showed obviously down‐regulated expression largely affecting AC (see below) as follows: *Wx^a‐dE^* (Endosperm‐box deleted in T1T2‐2 line), *Wx^a‐dU^* (unknown element deleted in T3T4‐2), *Wx^a‐dA^* (A‐box deleted in T5T6‐5), *Wx^a‐dC1^*, *Wx^a‐dC2^* and *Wx^a‐dC3^* (CAAT‐box deleted in T7T8‐4, T7T8‐5 and T7T8‐6, respectively), and *Wx^dS1^*, *Wx^dS2^* and *Wx^dS3^* (splicing‐site deleted/impaired in UISS‐1, UISS‐2 and UISS‐6) (Figure 1b). We selected a T10‐edited mutant (TFBg) for analyses.

To investigate the splicing patterns of the 5’UISS‐edited lines, we performed reverse transcription (RT)‐PCR and cDNA‐sequencing. UISS‐1 (*Wx^dS1^*), UISS‐2 (*Wx^dS2^*) and UISS‐6 (*Wx^dS3^*) generated multiple alternatively or atypically spliced transcripts with various frequencies, similar to *Wx^b^* in a *japonica* variety KY131 (Figure 1c). Three transcripts (*Wx^dS1^*‐3, *Wx^dS3^*‐4 and *Wx^b^*‐4) were found to retain the non‐spliced intron. These major alternative splicing events with size differences were confirmed by gel electrophoresis (Figure 1d). Obviously, the splicing‐site deletion in UISS‐1 and UISS‐6 resulted in the altered intron‐splicing patterns (and suppressed splicing of some transcripts). However, the 2‐bp deletion near the 5’UISS in UISS‐2 also produced an alternative transcript (*Wx^dS2^*‐1 with 58.3%) (Figure 1c), suggesting that this 2‐bp deletion might change the pre‐mRNA conformation affecting correct intron splicing.

Then, we used quantitative RT‐PCR (qRT‐PCR) to measure mature mRNA levels of these lines in developing endosperm. In T7T8‐4 (*Wx^a‐dC1^*), T7T8‐5 (*Wx^a‐dC2^*) and T7T8‐6 (*Wx^a‐dC3^*), the expression levels were down‐regulated to 37.4%, 32.7% and 24.9% of TFB, respectively (Figure 1e). The lines with deletions of the Endosperm‐box (T1T2‐2), unknown element(s) (T3T4‐2) and A‐box (T5T6‐5) also showed significantly decreased mRNA (85.2%, 67.4% and 60.5% of TFB, respectively). However, the rest lines with base variations at the targets had little or no expression changes. These results verified the regulatory roles of these putative CREs on transcription. In addition, all the 5’UISS‐edited lines exhibited decreased mRNA levels; especially, the lines *Wx^dS1^* (UISS‐1), *Wx^dS2^* (UISS‐2) and *Wx^dS3^* (UISS‐6) had only ca. 10% mRNA levels of TFB, suggesting that these mis‐splicing, atypical splicing and non‐splicing might largely reduce the transcript stability. The base editing of intronic splicing sites within coding regions could cause aberrant mRNA splicing and gene function knockout (Li *et al*., 2019). However, our strategy of editing exon/intron border sequences within 5’UTRs can quantitatively regulate gene activity and phenotypic performance (see below).

Next, we measured AC and GC of these lines. The lines without the CAAT‐box showed reduced ACs, from 24.6 % in TFB to moderate levels of 19.6% (T7T8‐4), 18.1% (T7T8‐5) and 17.8% (T7T8‐6) (Figure 1e). While ACs of UISS‐1, UISS‐2, UISS‐6 were 10.6%, 9.8% and 11.5%, respectively (Figure 1e); these lines are novel valuable ‘soft rice’ germplasms. TFBg had 2.4% AC, a typical glutinous rice. Some other edited lines also produced slightly reduced ACs, such as 23.8% (T3T4‐2), 22.8% (T5T6‐5) and 22.9% (UISS‐5). These AC variations were related to the corresponding *Wx* mRNA levels. In accordance with the AC reductions, T7T8‐4, T7T8‐5, T7T8‐6, UISS‐1, UISS‐2 and UISS‐6 showed increased GC values (62–83 mm, comparing to 56 mm of TFB) (Figure 1e). In addition, the polished grain appearances of T7T8‐4, T7T8‐5 and T7T8‐6 were similar to that of TFB (Figure 1f). However, due to the lower ACs in UISS‐1, UISS‐2 and UISS‐6, their polished grains had lower endosperm transparency with milky‐white appearances (Figure 1f).

We further used Rapid visco analysis (RVA) to assess the starch quality (Fitzgerald *et al*., 2003). The RVA viscosity indexes of the edited lines varied to various degrees relating to their ACs (Figure 1g). Among them, PT7T8‐4, PT7T8‐5 and PT7T8‐6 showed significantly decreased viscosity indexes, closer to those of *indica* HHZ (carrying *Wx^b^* with 17.1% AC) that has high grain quality.

Finally, we observed that the major agronomic traits (1000‐grain weight, grain length, grain width, plant height and plant morphology) of these edited lines were similar to TFB, except for slightly decreased 1000‐grain weight in UISS‐1, UISS‐2 and UISS‐6 (93%–95% of TFB).

In summary, we developed high‐efficient CRISPR/Cas9‐mediated promoter/5’UISS‐engineering strategies for generating new quantitative trait alleles with fine‐tuned transcriptional and post‐transcriptional regulations of gene expression activity. We expect that application of these grain‐improved lines having desirable AC and GC levels and their exploitation in hybrid rice breeding will provide rice products with better quality to meet consumer’s preferences. As CREs and 5’UTR introns are present in many genes, our study provides a promising breeding method for improvement of important traits in crops and other organisms.

## Conflict of interest

The authors declare no conflict of interest.

## Author contributions

Y.‐G.L. and Q.Z. designed the studies. D.Z., T.L., X.M., B.W., Z.Z., Y.Z., X.X., B.Y. and Z. Z. performed the experiments. D.Z., T.L., Q.Z. and Y.‐G.L. analysed data. Y.‐G.L. and Q.Z. wrote the paper.
